# Transhumeral loading during advanced upper extremity activities of daily living

**DOI:** 10.1371/journal.pone.0189418

**Published:** 2017-12-19

**Authors:** Alex J. Drew, Morgan T. Izykowski, Kent N. Bachus, Heath B. Henninger, K. Bo Foreman

**Affiliations:** 1 Department of Orthopaedics, University of Utah, Salt Lake City, Utah, United States of America; 2 Department of Bioengineering, University of Utah, Salt Lake City, Utah, United States of America; 3 Department of Veterans Affairs, Salt Lake City, Utah, United States of America; 4 Department of Physical Therapy and Athletic Training, University of Utah, Salt Lake City, Utah, United States of America; The Ohio State University, UNITED STATES

## Abstract

Percutaneous osseointegrated (OI) implants for direct skeletal attachment of upper extremity prosthetics represent an alternative to traditional socket suspension that may yield improved patient function and satisfaction. This is especially true in high-level, transhumeral amputees where prosthetic fitting is challenging and abandonment rates remain high. However, maintaining mechanical integrity of the bone-implant interface is crucial for safe clinical introduction of this technology. The collection of population data on the transhumeral loading environment will aid in the design of compliance and overload protection devices that mitigate the risk of periprosthetic fracture. We collected marker-based upper extremity kinematic data from non-amputee volunteers during advanced activities of daily living (AADLs) that applied dynamic loading to the humerus. Inverse dynamic analysis was applied to calculate the axial force, bending and torsional moments at three virtual amputation levels representing 25, 50, and 75% residual humeral length. The influences of amputation level, elbow flexion constraint, gender and anthropometric scaling were assessed. Results indicate that the proximal (25%) amputation level experienced significantly higher axial forces and bending moments across all subjects when compared to distal amputation levels (p≤0.030). Constraining elbow flexion had a limited influence on peak transhumeral loads. Male subjects experienced higher axial forces during all evaluated activities (p≤0.023). Peak axial force for all activities occurred during jumping jacks (174.5N). Peak bending (57.6Nm) and torsional (57.2Nm) moments occurred during jumping jacks and rapid internal humeral rotation, respectively. Calculated loads fall within the range of implant fixation failure loads reported in cadaveric investigations of humeral stem fixation; indicating that periprosthetic fracture may occur during non-contact AADLs. These kinematic data, collected over a range of AADLs, will aid in the development of overload protection devices and appropriate post-operative rehabilitation protocols that balance return to an active lifestyle with patient safety.

## 1. Introduction

One in five upper extremity amputees will abandon use of their prosthetic device. Among high-level, transhumeral amputees, the rate of abandonment nearly doubles [1, 2]. The primary reasons for device abandonment are poor fit, discomfort, pain, weight, and challenges associated with socket type prosthetic suspensions that rely on the soft tissues of the residual limb for fixation [2]. Percutaneous osseointegrated (OI) implants, which directly connect the prosthesis to the skeleton of the residual limb, are being developed worldwide as an alternative to socket suspension. These implant systems may alleviate many of the problems associated with current upper extremity prosthetics [3, 4]. However, maintaining the mechanical integrity of the bone-implant interface is crucial for safe clinical introduction of this technology.

European trials of percutaneous OI fixation in individuals with transhumeral amputation have employed torsional overload protection devices, and strict post-operative activity restrictions, to reduce the risk of periprosthetic fracture [3]. Yet only Welke et al. have investigated the ultimate bending fracture load of a percutaneous OI device implanted in the diaphysis of cadaveric humeri [5]. Unfortunately, the forces and moments that the humerus withstands during daily activities are unknown. A gap exists in the literature of upper extremity dynamics where investigations focus on either elite athletic activities, such as baseball pitching, or low load activities of daily living (ADLs) that typically consist of light lifting and personal hygiene tasks [6–9]. Relatively little information is available on the loads experienced by the humerus during moderate demand activities representative of an active amputee population. Collectively the moderate demand tasks explored in this investigation will be referred to as advanced activities of daily living (AADLs, section 2b).

Therefore, the purpose of this study was to collect upper extremity kinematic data during AADLs that apply dynamic loads to the humerus. To accomplish this, VICON motion capture data were collected from non-amputee volunteers (section 2a) so as to capture the kinetic influence of an integrated elbow and forearm. Inverse dynamic analysis was then used to calculate the forces experienced by the humerus at three virtual amputation levels (section 2c). Results were analyzed to determine the influence of amputation level (section 3b), constraining elbow flexion (3c), gender and anthropometrics (3d), and activity (3e) on the peak moments and forces experienced by the humerus. These data may be used to advance the design of upper extremity overload protection systems, and inform percutaneous OI rehabilitation protocols, that maximize return to function while protecting the critical bone-implant interface (section 4).

## 2. Materials and methods

### a. Subjects

Written informed consent was obtained from 40 healthy individuals (20 male, 20 female, median 28 years, range 19–56 years) to participate in this University of Utah Institutional Review Board approved study (IRB 00089237). Inclusion criteria required right arm dominance and the ability to complete the planned activities. Individuals with a history of shoulder surgery, or musculoskeletal injury limiting upper extremity function, were excluded. Anthropometric data, including: subject height, weight, axilla-to-acromion depth, and hand thickness at the 3^rd^ metacarpal head were collected for each subject.

### b. Advanced activities of daily living

Seven AADLs were analyzed (Table 1). Activities were selected based on review of high demand tasks captured in the Disabilities of the Arm, Shoulder and Hand (DASH) [10], Simple Shoulder Test (SST) [11] and American Shoulder and Elbow Surgeons (ASES) [12]score clinical surveys. Jumping jacks and rapid internal humeral rotation were also selected for their potential to apply high dynamic bending and torsional loads at the bone-implant interface. During passive elbow fall, the subject allowed their forearm to travel from a fully flexed to a fully extended position under the influence of gravity. This activity was selected to simulate the sudden release of the elbow locking mechanism found in many upper extremity prostheses, and the associated shock load that would be experienced at full elbow extension. To mimic the current functional capabilities of upper extremity prosthetics with locking elbows, three activities (jumping jacks, jogging, and tossing a ball underhand) were repeated with the elbow fixed at either 90 or 135 degrees of flexion using a rigid aluminum brace. Participants performed three trials of each activity.

**Table 1:** List of advanced AADLs ranked in ascending order of demand.

	Activity Description	Elbow Constraints
Low demand↓High demand	Elbow Fall	None
	Underhand Toss	None, 135°
	Jogging	None, 90°
	Briefcase (4.5kg) Carry	135°
	Internal Rotation	90°
	Jug Lift (1 gallon)	None
	Jumping Jacks (N = 3)	None, 135°

### c. Motion capture and data processing

**Fig 1:**
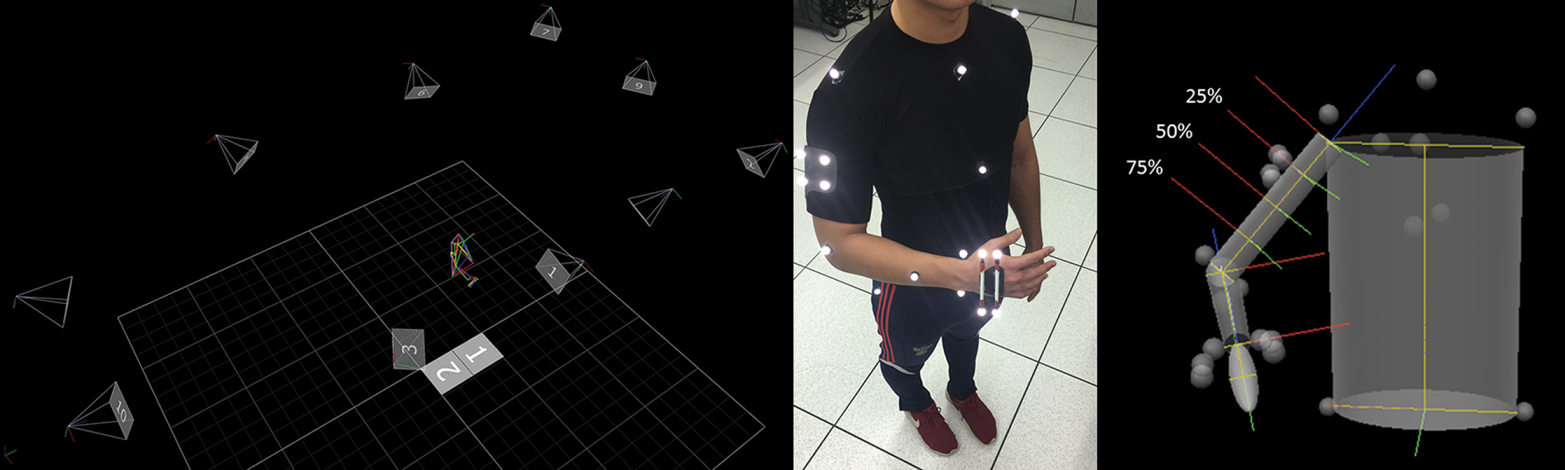
Capture volume and marker placement for 3D kinematic motion capture. Representative image of the experimental setup including 10 camera positions (pyramids), in-ground force plates (labeled 1 & 2 but not utilized), and kinematic model within the motion capture laboratory (left), marker placement defining rigid body segments (center), and Visual 3D upper extremity model including marker visualization, virtual amputation levels (25, 50, 75% residual humerus), segment geometries and coordinate axes (right).

Subject motion was captured using a 10-camera VICON Motion Analysis system (Vicon Motion Systems Ltd., Oxford, UK) in the Motion Capture Core Facility at the University of Utah Department of Physical Therapy and Athletic Training (Fig 1, left). Reflective markers were placed on each participant by palpating bony landmarks to define rigid segments of the trunk, right arm, forearm, and hand (Fig 1, middle). Fixed clusters of 4 markers each were placed on the arm and hand to increase visibility by overhead cameras. The use of fixed clusters also decreased skin motion artifact during tracking. Markers placed at the radial and ulnar styloid and distal posterior surface of the forearm were used to track the forearm segment. Reflective marker trajectories were recorded using VICON Nexus 2.3 (Vicon Motion Systems Ltd., Oxford, UK) software at an acquisition rate of 200Hz. Gaps in trajectories were first filled with the VICON Woltring filter (up to a maximum gap of 5 frames), and remaining gaps were manually filled using VICON spline, pattern, rigid body, and kinematic interpolation algorithms. The entire motion capture data set are available for download from the following link: https://doi.org/10.5281/zenodo.1040453.

Trajectory data were imported into Visual 3D (v5, C-Motion; Germantown, MD) and filtered with a low-pass Butterworth filter with a cut-off frequency of 6Hz based on residual analysis [13]. As is common practice in clinical biomechanics, the pose of each arm segment and the orientation of each joint axis was determined directly from the positions of the reflective markers on the arm. At each instant in time, the geometry of the arm and its joints were treated as a rigid-body mechanism in which each segment had 6 DOF, while the elbow and wrist joints were described using constraint equations. Separate models for activities involving external objects (briefcase carry, jug lift) were used to standardize these objects across all subjects. Both the one-gallon water jug and the briefcase were instrumented with markers to form their own rigid bodies for tracking. In both models, the linkage between the hand and external object was defined as a joint with six degrees of freedom.

All models were scaled to each subject using anthropometric data according to Dempster’s body segment parameters (BSPs) as reported by Winter [13]. The shoulder joint center was determined by constant inferior offset of the acromion marker by half of the axilla-to-acromion depth, similar to Rab et al. [14]. The elbow joint center was determined as the midpoint between the lateral and medial epicondyle markers. The wrist joint center was determined as the midpoint of the radial and ulnar styloid. Virtual amputation levels of the humerus were built into each model by creating joints with zero degrees of freedom located at 25% (proximal), 50% (mid-shaft) and 75% (distal) residual humerus length (Fig 1, right). The total mass of the arm above the elbow was divided equally among the four virtual segments.

As a matter of note, secondary analyses indicated that Winter’s BSPs provided more conservative estimations of forces and moments than alternate parameter sets proposed by de Leva et al. [15], which are based on anthropometrics defined by Zatsiorsky and Seluyanov [16]. These differences arise due to the male/female distribution within the respective populations (Winter’s data is all male), and the relative sizing of the individuals in the subgroups. As the motivation for the present study was to evaluate maximal forces and moments we felt it was appropriate to utilize Winter's BSPs as they produce the most conservative results.

Model-based reaction moments and forces were calculated at the three virtual amputation levels using the 'model_based_data_computation' function within Visual 3D. Based on residual analysis, these calculations were filtered with a low-pass Butterworth filter at 10Hz [13]. Event processing tools within the Visual 3D software environment allowed for extraction of peak load events for each trial. Peak moments and forces for each subject were determined by averaging peaks from three trials of each activity. Peak bending moments were calculated as the resultant of the moments in the anteroposterior and mediolateral anatomic planes averaged over three trials.

### d. Statistical analyses

Statistical comparisons were carried out using 2-tailed independent t-tests assuming unequal variances. In cases where repeated measures were made (e.g. free vs. constrained elbows) paired t-tests were used. Paired t-tests were also used for within subject comparisons (e.g. virtual amputation level). Significance levels were set at p≤0.05 for all comparisons.

## 3. Results

### a. Subjects

No significant difference was found between the ages of male and female subjects (p = 0.121). Anthropometric data indicated that male subjects were significantly larger than female for all collected measures (Table 2).

**Table 2:** Subject demographics and anthropometric measurements.

	Age (years)	Mass (Kg)	Height (cm)	Axilla Depth (cm)	Hand Thickness (cm)	Hand and Forearm, Combined Mass (kg)
Subjects (n = 40)	28 ±7	70.3 ± 16.5	174.2 ± 9.7	10.4 ± 1.3	2.8 ± 0.3	1.6 ± 0.4
Male (n = 20)	30 ± 9	82.3 ± 14.1	181.1 ±7.2	11.0 ± 1.4	3.0 ± 0.2	1.8 ± 0.3
Female (n = 20)	26 ±5	58.4 ± 7.6	167.0 ± 5.9	9.7 ± 0.8	2.6 ± 0.2	1.3 ± 0.2
p-value	0.121	*≤0*.*001*	*≤0*.*001*	.*0002*	*≤0*.*001*	*≤0*.*001*

Mean ± standard deviation.

### b. Amputation level

**Fig 2:**
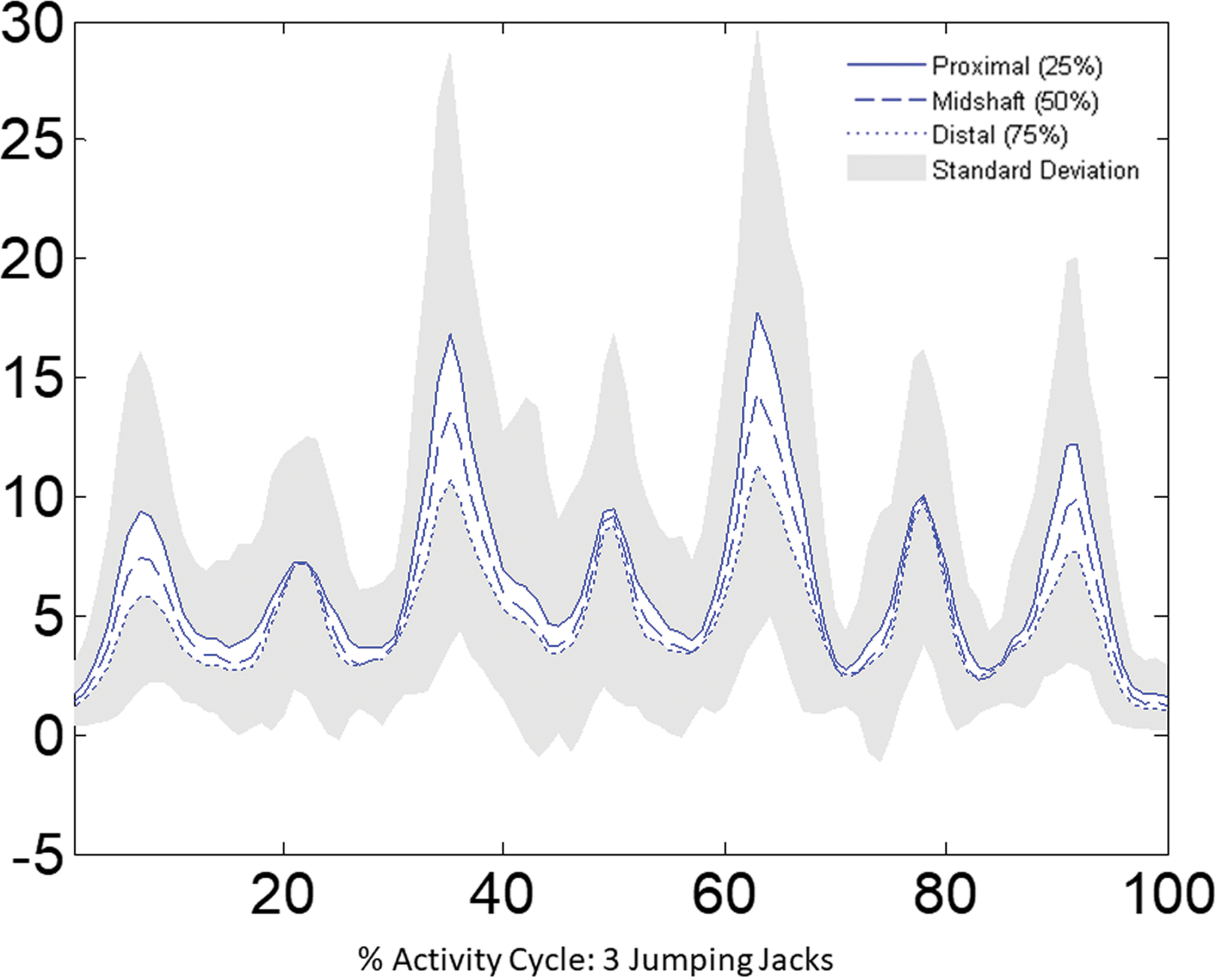
Bending moments during jumping jacks. The proximal humerus (25% amputation level) experienced the highest bending moment across all subjects. Lines represent averages across all subjects for each amputation level. Shaded areas represent upper and lower standard deviations for the proximal and distal humeral segments, respectively.

Peak bending moments and axial forces across all subjects showed that the 25% amputation level consistently experienced higher peak axial forces and bending moments than the 50% amputation level (p≤0.030) for all AADLs with the exception of jogging. Jogging comparisons were restricted to subjects with a full captured gait cycle for both the free and constrained elbow condition (n = 15). Likewise, the 50% amputation level consistently experienced higher peaks than the 75% amputation level (p≤0.023). This is illustrated by the representative mean bending moment curves for each humeral amputation level during jumping jacks (Fig 2). No relationship was observed between amputation level and torsional loading. This was anticipated as amputation segments were assumed to be coaxial, resulting in uniform torsional loading independent of virtual amputation level. Since the success of overload protection devices and conservative rehabilitation strategies relies on understanding maximal loading conditions, subsequent results focus on the proximal (25%) amputation level. Additionally, the anticipated risk of periprosthetic fracture is greatest in this proximal region where cortical bone is thinnest.

### c. Effect of the elbow flexion constraint on loading

**Fig 3:**
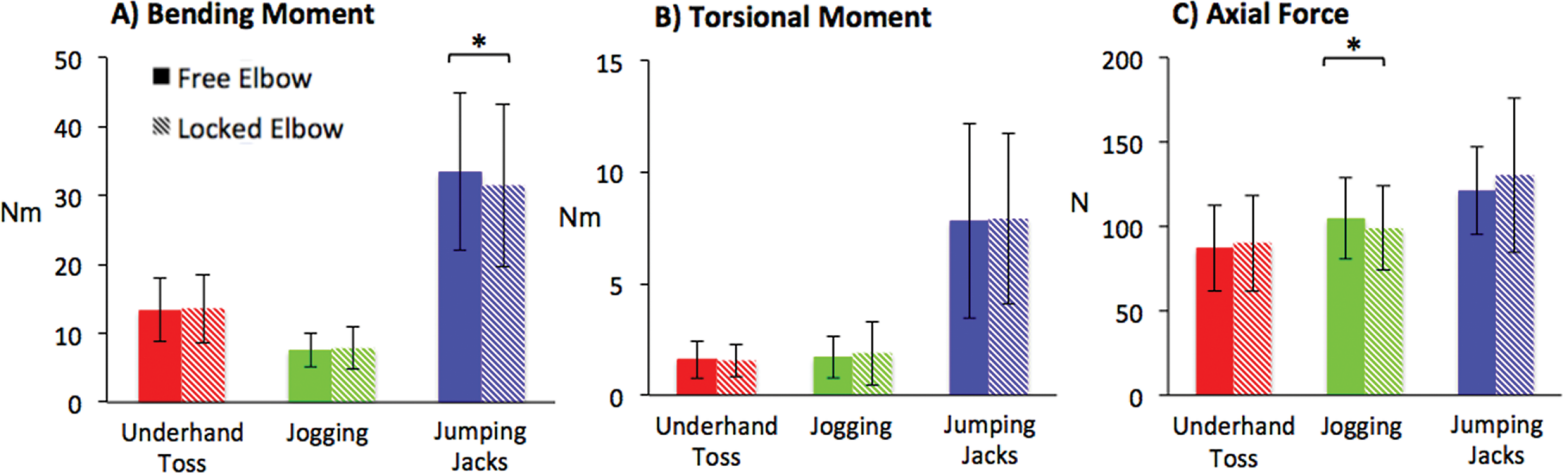
Comparison of free and locked elbow conditions. Peak moments and forces at 25% humeral length for AADLs performed with both free and locked elbow: underhand toss, jogging, jumping jacks. (A) Significant differences were only observed for peak bending moments during jumping jacks (p = 0.037). (B) No significant differences in torsional moment. (C) Significant differences were only observed for peak axial forces during jogging (p = 0.017). Note that jogging included only N = 15 subjects for which a full gait cycle was available in both free and constrained elbow conditions.

Of the three AADLs performed with both a free and locked elbow (underhand toss, jogging, and jumping jacks), only the peak bending moment during jumping jacks and the peak axial force during jogging showed a significant difference between elbow conditions (Fig 3). No significant difference was observed between elbow conditions for torsional moment. Since only 2 of 9 comparisons between free and locked elbows showed a significant difference, and the differences in magnitudes were small (<10% of mean) only the free elbow condition is presented subsequently.

### d. Effects of gender / anthropometrics

**Fig 4:**
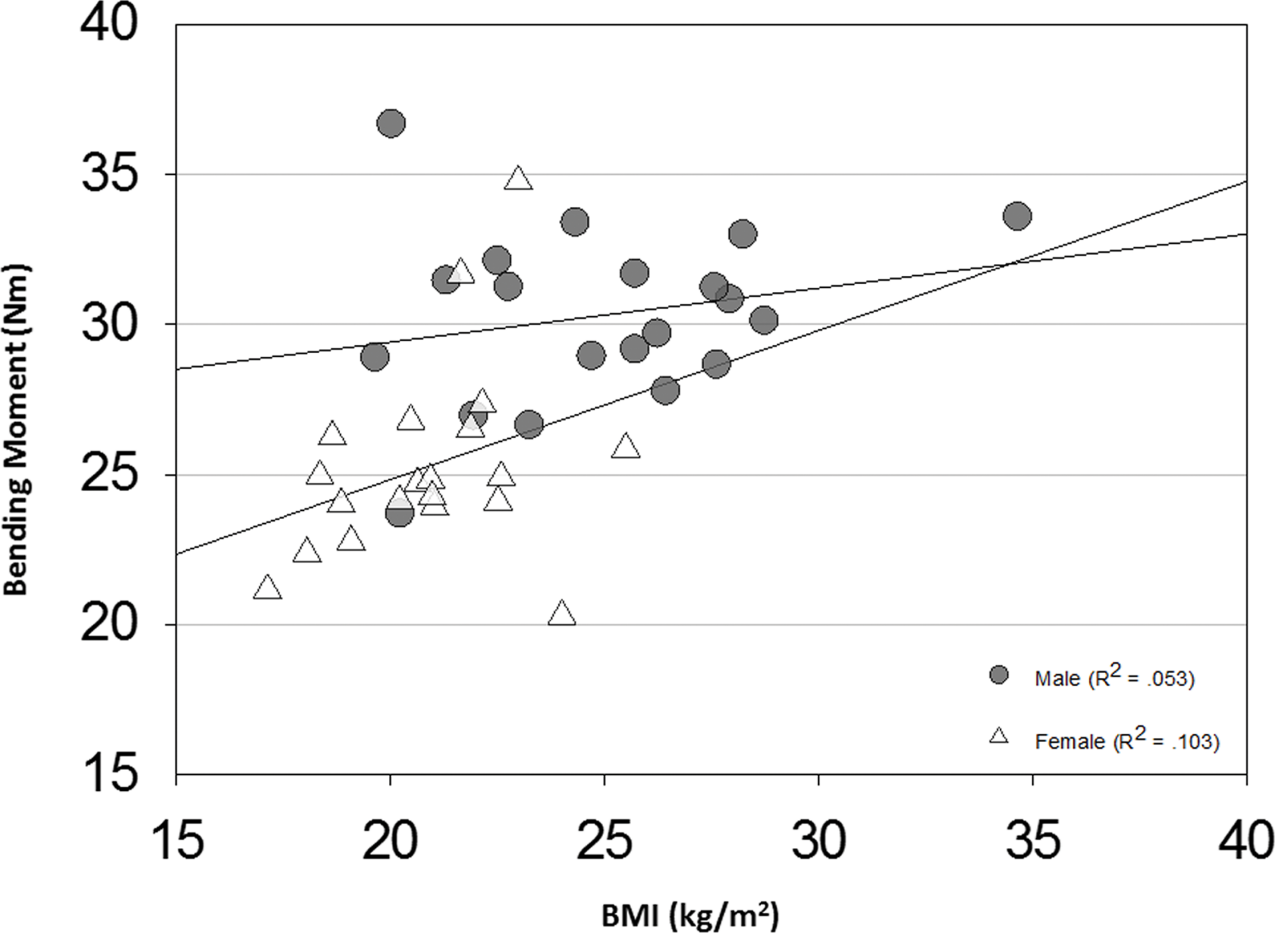
Male and female bending moments during jug lifting. Peak bending moments by gender during the jug lift AADL can be attributed to differences in anthropometric measurements, since all subjects lifted the same object. Peaks are presented for the proximal (25%) humeral segment. Male peaks were significantly greater than female (p<0.001).

Average peak axial forces at the (25%) proximal level were greater in males than females for all AADLs (Table 3). Males experienced higher bending moments during elbow fall, internal rotation, jug lift, and jumping jacks. They also experienced increased torsional moments during elbow fall, jogging, internal rotation, and jumping jacks. Gender differences are most clearly demonstrated in the jug lifting activity where the effects of body size on pure bending moment are most isolated (Fig 4).

**Table 3:** Gender differences by AADL at the 25% amputation level.

	Bending Moment (Nm)			Torsional Moment (Nm)			Axial Force (N)		
AADL	Male	Female	P-value	Male	Female	P-value	Male	Female	P-value
Elbow Fall	8.3 ± 3.0	5.5 ± 2.1	*0*.*002*	1.0 ± 0.5	0.5 ± 0.2	*0*.*002*	68.2 ± 11.3	47.7 ± 8.3	*≤0*.*001*
Underhand Toss	14.4 ± 3.4	12.3 ± 5.4	0.173	1.8 ± 0.5	1.4 ± 1.0	0.188	98.4 ± 22.4	75.8 ± 22.9	*0*.*004*
Jogging	8.6 ± 2.4	7.5 ± 2.3	0.292	2.1 ± 0.9	1.2 ± 0.4	*0*.*010*	116.7 ± 24.1	94.1 ± 17.7	*0*.*023*
Briefcase Carry	11.4 ± 2.6	10.3 ± 2.2	0.184	3.1 ± 1.0	2.7 ± 0.7	0.209	113.8 ± 12.3	97.3 ± 8.8	*≤0*.*001*
Internal Rotation	23.5 ± 8.5	15.0 ± 5.7	*0*.*001*	24.9 ± 11.2	12.8 ± 6.1	*≤0*.*001*	52.0 ± 15.0	37.5 ± 7.6	*0*.*001*
Jug Lift	30.3 ± 2.8	25.3 ± 3.2	*≤0*.*001*	5.4 ± 2.0	4.3 ± 1.6	0.067	87.8 ± 9.1	74.2 ± 7.6	*≤0*.*001*
Jumping Jacks	40.7 ± 9.4	26.2 ± 8.1	*≤0*.*001*	10.7 ± 4.0	5.0 ± 2.4	*≤0*.*001*	138.7 ± 21.4	103.8 ± 16.9	*≤0*.*001*

### e. Moments and forces by advanced AADL

**Fig 5:**
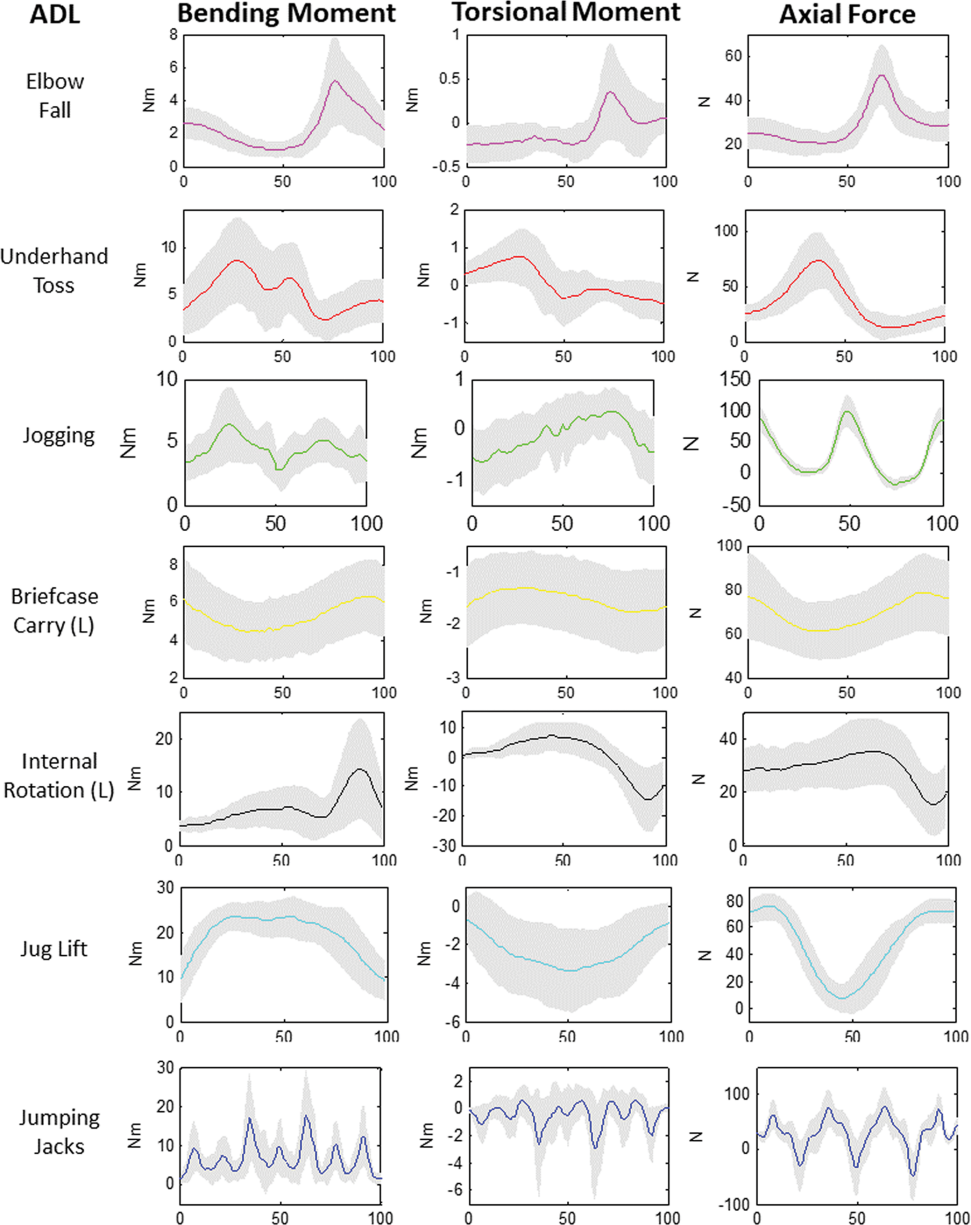
Mean bending moment, torsional moment and axial force curves. Mean (solid line) ± SD (shaded) moments and forces at 25% humeral length for advanced AADLs as a percent of the activity cycle (x-axis). Jogging curves are from subject data that captured a full gait cycle (N = 23). Jumping jack curves represent three consecutive jumping jacks constituting one cycle.

The highest peak bending moments and axial forces occurred during jumping jacks, and the highest peak torsional moments occurred during internal rotation activities (Fig 5, Table 4). Due to camera capture volume constraints, and variable self-selected jogging speed between subjects, data collection of an entire gait cycle was not possible in some subjects. As a result, jogging curves were generated from a subset of 23 subjects (12 male, 11 female) for which a full gait cycle was captured in the free elbow condition.

**Table 4:** The highest peak moments and forces in advanced AADLs.

Metric	AADL	Max
Bending Moment	Jumping Jacks	57.6 Nm
Torsional Moment	Internal Rotation	57.2 Nm
Axial Force	Jumping Jacks	174.5 N

## 4. Discussion

In this study we calculated the reaction forces and moments at three virtual transhumeral amputation levels using data from healthy adult subjects. Amputation levels representing 25, 50 and 75% of the residual humerus length were quantified using marker-based motion capture and inverse dynamic analysis. Results indicate bending moments and axial forces were consistently highest at the most proximal (25%) amputation level. Increased loading can be attributed to the longer moment arm and mass distal to the virtual amputation site. Torsional loading was unaffected by the virtual amputation level.

The influence of segment length and inertia is further illustrated when comparing male and female loading. Males were statistically taller with higher body mass in this cohort, and experienced higher axial forces for all examined activities (Table 3). Similar to the influence of amputation level on the magnitude of calculated loads, this result is unremarkable given the increased mass distal to the amputation site in males. Notably, this significant increase in the male axial force does not hold for bending and torsional moments across all activities. Only activities that created a maximal excursion of distal arm segment (forearm and hand) centers of mass away from the humeral axis, thereby maximizing the moment arm, exhibited a statistically significant difference between males and females. These activities included elbow fall, internal rotation, lifting a gallon water jug to shoulder height and jumping jacks. Activities with loosely defined motion profiles that were performed at a self-selected speed, such as tossing a ball underhand, jogging, and carrying a briefcase, did not exhibit significant differences in peak bending or torsional loads between males and females.

The impact of the elbow flexion constraint on the magnitude of dynamic loads was investigated during underhand toss, jogging, and jumping jacks. A constrained elbow condition was applied to these activities due to the inability of current prosthetic technologies to mimic coordinated elbow motion during high-speed tasks. A common compensatory strategy would involve locking the prosthetic elbow at a desired degree of flexion for a given task, which could influence the dynamics of the motion. Significant differences between a constrained and free elbow condition were only observed in 2 of 9 tested conditions. In all cases the magnitude of the difference was <10% of the mean. For consistency of comparison between all tested AADLs, the free elbow data was used for further analyses.

Previous kinematic investigations in the upper extremity have focused on elite, high demand activities such as baseball pitching or low demand reaching and hygiene tasks, making direct comparisons of the present data difficult [6–8, 17]. As a result, it is more useful to compare the loads herein to biomechanical investigations of periprosthetic fracture in the humerus. Welke et al. has performed the only investigation that has examined fracture loads of a percutaneous OI stem placed in the diaphysis of cadaveric humeri [5]. In that investigation, the mean±SD bending failure load was found to be 36.7±11.0Nm. This places average peak calculated bending loads for AADLs in this study (range: 5.5–40.7 Nm) within the range of periprosthetic failure. Comparison of calculated average peak torsion loads in the present study (range: 0.5–24.9 Nm) to investigations of periprosthetic fracture of shoulder and elbow arthroplasty stems lead to similar observations, with reported periprosthetic fracture loads ranging from 5.3–23.4 Nm at the shoulder [18] and 16.5–79.3 Nm at the elbow [19].

While low failure loads observed in biomechanical investigations may be attributed to the advanced age and poor bone quality of specimens, this likely increases their applicability to the upper extremity amputee population where disuse osteoporosis due to the reduction of skeletal loading can lead to adverse cortical thinning and reduced material properties [20–23]. Absence of bone ingrowth in cadaveric studies may also contribute to reduced failure loads but may be indicative of the acute rehabilitation period following implantation of percutaneous OI devices when osseointegration is minimal. Based on these observations, protective strategies should be employed to shield the bone-implant interface from damaging loads in upper extremity amputees with percutaneous OI devices. Additionally, implant designs should seek adequate initial stability to tolerate planned post-operative rehabilitation strategies.

In a series of 16 transhumeral patients treated with the OPRA implant system (Integrum, Sweden) the protective strategy was two-fold. First, a six month healing period was observed following implantation to allow for osseointegration of the system. Upon commencement of loading, a lightweight training prosthesis was used to slowly acclimate the patient to gradually increasing loads over a 12-week period. Second, a rotational safety device was employed to prevent high torsional moments at the bone-implant interface. This device could be adjusted to increase or decrease maximum torque transmission based on patient specific needs [3]. This overload protection strategy, while focused on a single failure mechanism (torsion), can be adapted to address the inverse relationship shown in this investigation between reported upper extremity fracture loads and the reaction forces and moments based on amputation level (i.e. fracture loads decrease in the proximal humerus while loading increases).

The present study is subject to limitations. The primary limitation is the use of a non-amputee population for analysis. This decision allowed for a greater recruitment population and a more robust analysis of the range of loading experienced in the intact humerus. While it disregards the inherently modified kinematics in the amputee population, the speed and range of motion in healthy volunteers is an appropriate surrogate for peak functional outcomes that can be expected from a prosthetic user. Mass characteristics of upper extremity prosthetics were also disregard. The authors recognize that the choice of a lightweight cosmesis, body powered, powered, or hybrid upper extremity prosthetic would impact loading at the bone-implant interface. We also recognize that amputees may be fit with multiple prosthetic options for specific activities. Finally, the limited number of AADLs in this study only begins to capture the range of activities pursued by an active amputee population. It is expected that as percutaneous OI patients adapt to their newfound range of motion and arm control strategies that they will become involved in higher demand activities that may exceed those evaluated here. This is, in turn, further motivation for the development for overload protection devices for transhumeral amputees undergoing OI.

## 5. Conclusions

Percutaneous OI devices hold tremendous promise for the upper extremity amputee population, which continues to experience high prosthetic rejection rates. Axial forces, and bending moments, at the proximal (25%) amputation level exceed those at the 50 and 75% amputation levels. Peak axial forces in this study occurred during jumping jacks (174.5 N). Peak bending (57.6 Nm) and torsional (57.2 Nm) moments occurred during jumping jacks and rapid internal humeral rotation, respectively. Results of this study indicate that the risk for fracture at the bone-implant interface exists during non-contact AADLs, highlighting the need for quantification of the loading environment of the amputated humerus during a range of activities. The data presented in this study, when coupled with biomechanical investigations of implant fixation, will inform the design of overload protection devices that appropriately balance return to an active lifestyle with the risk of percutaneous OI device overload.

## Supporting information

S1 Appendix Upper extremity joint reaction forces and moments. Complete subject data, including: demographics, anthropometric measures, axial forces, bending and torsional moments. Load values reported at shoulder, elbow, and three virtual amputation levels. Note that shoulder and elbow metrics were not reported in the manuscript as they were not relevant to the question of forces experienced by transhumeral amputee populations.
(XLSX)
